# Memory and False Memory for Information That Is Either Expected or Unexpected Based on Age Stereotypes

**DOI:** 10.5964/ejop.13729

**Published:** 2025-02-28

**Authors:** Z. Asude Kaymak Gülseren, Simay İkier

**Affiliations:** 1 Department of Psychology, Bahçeşehir University, İstanbul, Türkiye; 2 Faculty of Human and Social Sciences, Department of Psychology, Marmara University, İstanbul, Türkiye; Glasgow Caledonian University, Glasgow, United Kingdom

**Keywords:** aging, stereotype, expectation, memory, recall, recognition, false memory

## Abstract

Age is a major social categorization information because it is one of the first attributes that is perceived about an individual. The present study used the misinformation paradigm to investigate memory and false memory for information that is either expected or unexpected based on age stereotypes. Young adults were presented with a passage depicting a crime. The passage also contained information about the physical performance and social behavior of the main character that was either expected (expected information condition) or unexpected (unexpected information condition) for his age. The main character was a young adult in the expected information condition and an older adult in the unexpected information condition. Next, misinformation was provided about a detail related to the crime. After a non-verbal filler task, participants recalled the exact sentences from the passage, and then they completed a forced-choice recognition test for them. Measures of attitudes toward older adults did not differ across the groups. The results revealed worse recognition memory for the sentences and higher false recognition of the misinformation in the expected information condition than in the unexpected information condition. The recall test revealed higher commission errors in the expected information condition than in the unexpected information condition. Commission errors were in general consistent with the information in the passage. The results imply that stereotypically expected information is automatically processed, making it more vulnerable to memory errors. The study contributes to the understanding of the memory processes underlying stereotyping that can lead to prejudice and discrimination.

One of the main attributes by which society perceives and forms impressions of individuals is their age. In most cases, the perception of the age of the individual activates stereotypes, i.e., beliefs, knowledge, and expectations about that age group (Hamilton & Sherman, 1994). In turn, stereotypes affect how the individual is categorized and socially judged (Montepare & Zebrowitz, 1998). Unfortunately, negative aging stereotypes are dominant (e.g., Kite et al., 2005; Kite & Johnson, 1988). Negative stereotypes of aging can create stereotype threat, hindering older individuals’ physical and cognitive performance (Levy & Leifheit-Limson, 2009), as well as their well-being (Chasteen & Cary, 2015). As a result, the way that older individuals are perceived in society is a major determinant of their quality of life and performance, as well as their mental health (Levy et al., 2021). Investigating the memory processes that underlie access to stereotypical information is important to understand its persistence and vulnerability to errors. In the present study, we are specifically interested in memory performance and false memory for information that is either expected or unexpected based on age stereotypes.

Initial views on memory for stereotypical information state that individuals search for information that fits their schemas, i.e., their generic knowledge about the world, and remember such information better (Bartlett, 1932). Stereotypes are instances of schemas in which the generic knowledge is about a social group (e.g., Macrae et al., 1994). In turn, based on prior knowledge, individuals create common expectations about that social group.

While the information that is expected based on stereotypes can sometimes aid memory by filling in the missing details with the most probable ones, it can also impair memory by leading to inaccurate assumptions. The script pointer plus tag (SP + T) model (Woll & Graesser, 1982) proposes that information that is expected based on stereotypes is coded as a merely generic example of what is expected, whereas information that is unexpected needs to have a unique tag because it violates expectations. This model is supported by evidence showing more accurate retrieval of information that is schematically expected than unexpected (e.g., Neuschatz et al., 2002).

Research also shows that information that is expected based on schemas and stereotypes can result in false memory (e.g., Brewer & Treyens, 1981; Charlton & Leov, 2021). In a milestone study, Sulin and Dooling (1974) showed that when a protagonist’s name activated prior knowledge that was not present in a passage, then there was false recognition of that knowledge. This reflects the automaticity of information processing when it is expected based on existing knowledge. Findings also showed more false memory and source misattributions for information that is expected based on stereotypes (Kleider et al., 2008). One reason for higher false memory for information that is expected based on stereotypes may be that it is retrieved as gist, without the specific details. Fuzzy-trace theory (Brainerd & Reyna, 2002) states that verbatim traces are precise representations of memory, while gist traces are meaning-oriented, summarized memory representations. Retrieving the gist of the information instead of the verbatim information increases the probability of memory errors. Given that information that is unexpected based on stereotypes receives more attention, it is reasonable to expect that such information would be retrieved in a more verbatim manner, resulting in a lower probability of false memories. On the other hand, if we process stereotypically expected information automatically, the reliance on the gist of information can result in memory errors even for stereotypically unrelated information.

When negative stereotypes outweigh the positive ones, it can result in ageism (Greenberg et al., 2004), and unfortunately, in meta-analyses (e.g., Kite et al., 2005; Kite & Johnson, 1988), it was shown that negative stereotypes of aging are more dominant. Indeed, negative aging stereotypes have become especially dominant in the last few years, reinforced by the Covid-19 pandemic (Monahan et al., 2020, but also see Carlson et al., 2020). The present study focused on the question of whether young adults’ memory performance and false memory are affected by whether the information is expected or unexpected based on age stereotypes. To our knowledge, this question has not been investigated in the literature yet. Investigation of the cognitive processes related to the automatic processing of information in memory due to the presence of age stereotypes is undeniably important since it can eventually lead to prejudice and discrimination. We investigated this question by using the misinformation paradigm (Loftus et al., 1978) in which the misinformation that is provided after the target information can lead to false memory for the target information. We especially focused on physical performance and social behavior that are expected for young adults, but not for older adults due to age stereotypes. We controlled for attitudes toward older adults considering that they might affect stereotypical expectations. Based on prior research and theoretical propositions (e.g., Woll & Graesser, 1982), we hypothesized better recall and recognition, and less vulnerability to false memory (Brainerd & Reyna, 2002) when the information is unexpected based on age stereotypes.

## Method

### Research Design

The study has a basic design with a single independent variable with two levels (information type: expected information condition, unexpected information condition). The dependent variables are memory accuracy and false memory in recall and recognition tests.

### Participants

Eight participants were excluded because they stated that they did not hear the first sentence of the passage from the voice recording that contained critical information about the main character’s age. The inclusion criteria were being healthy young adult university students, the exclusion criterion was not being a native speaker of Turkish. Participants were randomly assigned to the conditions in which the passages included either information that is expected (expected information condition) or unexpected (unexpected information condition) of the main character’s age. A total of 88 participants were recruited for the experiment. An equal number of participants were assigned to each condition, consisting of 33 males and 55 females within the age range of 18 and 28. In the expected information condition, there were 27 females and 17 males. In the unexpected information condition, there were 28 females and 16 males^1^1The sample size was determined based on similar prior research (e.g., Araya et al., 2003; Kleider et al., 2008).. Table 1 shows the minimum and maximum scores, means and standard deviations for age and the number of years of education.

**Table 1:** Descriptive Statistics for Age and Years of Education

	Expected Information Condition (*n* = 44)				Unexpected Information Condition (*n* = 44)			
Variable	*Min*	*Max*	*M*	*SD*	*Min*	*Max*	*M*	*SD*
Age	18	28	23.60	3.14	18	28	23.05	2.92
Years of Education	12	21	16.30	2.60	12	22	16.27	2.56

### Materials

The experimental procedure involved listening to a passage that either contained expected or unexpected information based on age stereotypes, responding to the misleading question about the passage, completing a non-verbal filler task, completing the recall and the recognition test for the information in the passage, responding to the manipulation check question about the passage, and finally filling in Kogan’s Attitude Toward Older People Scale (KAOP), Positive and Negative Ageism Scale (PNAS), and the demographic form. The subsequent section provides detailed information about the materials.

The voice recordings and the texts for the passages, the misleading question, and the questions for the recognition test are presented in Gülseren and İkier (2025)^2^2All materials, instructions, and their English versions are presented in the OSF repository in Gülseren and İkier (2025)..

#### The Passages

The passages in the two conditions were the same except that the first sentence stated that the main character is 20 years old in the expected information condition and 80 years old in the unexpected information condition. The passages described an attempted crime in which a snatcher advances a hand into the pocket of the main character’s friend, and the main character notices the incident and runs after the snatcher. The passages also described the habits (e.g., going for a run every morning) and some characteristics (e.g., energetic, sporty) of the main character and his plans for that day (e.g., going to a birthday party of a friend). While these physical and social characteristics were expected for a 20-year-old adult, they were unexpected for an 80-year-old adult based on the age stereotypes in the country in which the data were collected (Çayır, 2012; Danış & Kara, 2017).

Each passage contained eight sentences. The sentences were voice recorded to prevent reading them multiple times and to control possible differences in reading speed across participants. First, a single recording of the passage that included the first sentence for both conditions was created. Then, for the expected information condition, the first sentence for the unexpected information condition was cut out and for the unexpected information condition, the first sentence for the expected information condition was cut out. As a result, the recording for the rest of the sentences was the same for the two conditions. The speed of the utterance was reduced to an average speed and the sentence breaks were synchronized to eliminate tracking difficulties by using Adobe Premiere Pro (Adobe, 2003). In the final recording, each word was clearly heard, and the tone of voice was stable and clear, with no particular emphasis on any of the words. The duration for each recording is 44 seconds.

#### The Misleading Question

The misleading question was constructed by changing the type of hat the snatcher was wearing in the sentence that described the main character noticing the snatcher and by putting the sentence into question form.

#### Non-Verbal Filler Task

It is a four-minute task to that includes simple mathematical operations. The purpose of the filler task is to create a filled interval to prevent the rehearsal of the passage before the memory tests.

#### Recall Test

The test required verbatim recall in which participants were asked to retrieve the exact sentences from the passage in any order.

#### Recognition Test

It is a forced-choice recognition test for each sentence in the passage. Participants were asked to choose the exact sentence that was in the passage out of four choices. The incorrect choices were constructed by changing the details for the correct sentence. For the sentence that was used to provide misinformation, two of the incorrect choices included the misinformation.

#### Manipulation Check for The Passages

The question checks whether the information in the passage was expected or unexpected considering the main character’s age for the participants. Participants were asked to rate to what extent the characteristics and the behaviors of the main character in the passage were consistent with their expectations of characteristics and behaviors of that age group. The ratings were completed on a 5-point Likert scale ranging from “1 = Totally inconsistent” to “5 = Totally consistent”.

#### Kogan’s Attitude Toward Older People Scale (KAOP)

The scale contains 17 positive and 17 negative items measuring attitudes toward older adults (Kogan, 1961). While “*It is foolish to claim that wisdom comes with age,”* is an example of a negative item, “*People grow wiser with the coming of old age,”* is an example of positive item of the scale. The items are rated on a 6-point Likert scale ranging from “1 = Totally disagree” to “6 = Totally agree”. A total score of 102 is evaluated as a neutral attitude toward older adults and higher scores indicate more positive attitudes. The Cronbach’s alpha for the cultural standardization of the scale is .84 for the whole scale, and .77 and .79 respectively for positive and negative subscales (Erdemir et al., 2011).

#### Positive and Negative Ageism Scale (PNAS)

The scale is specifically designed to measure university students’ attitudes toward older individuals. The scale has two subscales measuring positively discriminative attitudes and negatively discriminative attitudes, consisting of 13 positive and 10 negative statements respectively. “*Elderly should withdraw from everyday life,*” is an example of a negative item of the scale and “*In everyday life, elderly should be given priority,”* is an example of a positive item. The statements are rated on a 5-point Likert scale, ranging from “1 = Totally disagree” to “5 = Totally agree”. Higher scores indicate more positively discriminative and less negatively discriminative attitudes toward older adults. The Cronbach’s alpha for the scale is .80 (Yurttaş & Sarıkoca, 2018).

#### Demographic Form

The form includes questions on age, sex, number of years of education, and general health of the participants.

### Procedure

**Figure 1:**
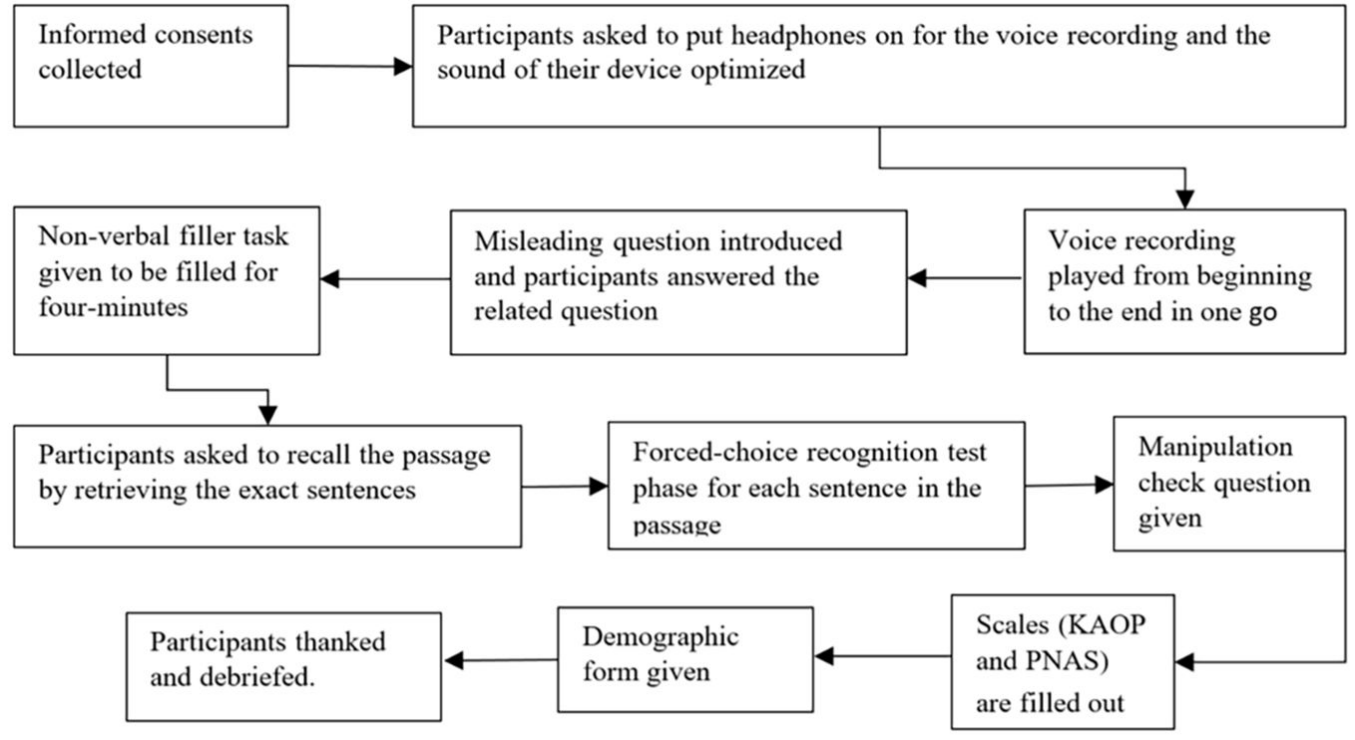
Flowchart for the Procedure

The study was approved by Bahçeşehir University Ethics Review Board. The study was conducted by having an online face-to-face meeting with each participant on the Zoom platform. A flowchart for the procedure is provided in Figure 1.

All participants provided informed consent. First, the voice recording for the passage was sent to the participants and the participants downloaded the recording to their computer to prevent problems related to the internet connection while listening to the passage. The participants were asked not to start the recording themselves. The experimenter started and ended the recording by accessing the participants’ screen for the duration of the recording.

All participants used headphones to eliminate external noise. First, participants were asked to put their headphones on. Then, the sound quality and level were checked by the experimenter with a short voice control recording that included only two words (i.e., “voice control”). The sound level was optimized to minimize information loss. Then the participants were asked to carefully listen to the passage without a break. They were told that once the voice recording starts, it cannot be paused or re-played. They were asked to remember the sentences in the passage for a subsequent test. Then, the recording was started and stopped at the end by the experimenter. Participants were asked to delete the voice recordings permanently from their computers after the recording was stopped.

Next, participants were given the misleading question and responded to the question as “yes” or “no”. Then, they were engaged in the non-verbal filler task in which they were asked to respond as accurately as possible. Then, participants completed the recall test and the recognition test. Afterward, participants responded to the manipulation check question. Finally, they filled out the KAOP, the PNAS, and the demographic form. All participants were debriefed.

### Data Analysis Plan

The results section starts with the descriptive statistics for the sample and a check for control variables. First, descriptive statistics for years of education and sex distribution were provided. Then, responses for the manipulation check question were compared with an independent measures *t*-test, to ensure control. Thereafter, KAOP and PNAS were analyzed and compared across conditions with an independent measures *t*-test. KAOP total score and PNAS positive and negative scores were correlated with memory measures (lenient recall, strict recall, verbatim recall, and recognition) by using Pearson correlation.

Then, the section continues with the tests of the main hypotheses of the study. Recall and recognition performance was assessed and compared across the conditions with independent measures *t*-test. A chi-square test was conducted to compare the two conditions for the generation of misinformation in the recall and the recognition tests.

Errors on the memory tests (omission, commission, and distortion) were assessed and compared across the conditions by using independent measures *t*-test. Lastly, the two conditions were compared in terms of the expected, unexpected, and unrelated commission errors by using independent measures *t*-test.

## Results

The data and the syntax for the analyses are available in Gülseren and İkier (2025).

### Participants

One participant’s datum was removed from the expected information condition because his z-score was above three in the recall test. The final sample that we conducted the analyses on consisted of 87 participants.

### Responses to the Manipulation Check Questions

Responses to the manipulation check questions showed that the characteristics and behaviors of the main character in the passage were more expected of his age in the expected information condition, *M* = 3.86, *SE* = 0.12 than in the unexpected information condition, *M* = 2.20, *SE* = 0.15, *t*(85) = 8.61, *p* < .001, *d* = 1.86.

### Attitudes Toward Older Adults

Participants in the two conditions did not differ in their attitudes toward older adults. For KAOP, *M* = 130.35, *SE* = 2.51 for the expected information condition, and *M* = 124.30, *SE* = 2.68 for the unexpected information condition, *t*(85) = 1.65, *p* = .103, *d* = 0.35. For, PNAS positive subscale *M* = 42.28, *SE* = 0.81 for the expected information condition, *M* = 42.05, *SE* = 1.19 for the unexpected information condition, *t*(85) = 0.16, *p* = .872, *d* = 0.03. For PNAS negative subscale, *M* = 41.07, *SE* = 0.84 for the expected information condition, and *M* = 39.41, *SE* = 0.97 for the unexpected information condition, *t*(85) = 1.30, *p* = .20, *d* = 0.28.

Based on the scores for KAOP and PNAS, our participants in general had relatively positive attitudes toward older adults. Exploratory analyses showed that the attitude scores did not correlate with any of the memory measurements, all *p*’s > .05.

### Scoring for the Recall Test

Recall scores were calculated by three different scoring criteria: lenient criterion, strict criterion, and verbatim criterion. Two independent raters scored the recalled information based on these criteria. Passages were broken down into idea units, which is a widely used procedure in prose memory research (e.g., Bransford & Johnson, 1972). The resulting 58 idea units were designated based on semantic units such as subjects, adjectives, propositions, and verbs which corresponded to either single words or phrases (Bransford & Johnson, 1972). The idea units are presented in Gülseren and İkier (2025).

For the lenient criterion scoring, a flexible approach was taken in which synonyms and analogies of the idea units that conveyed the same meaning, different conjugations of the correct verbs, and changes in the location of the idea units were counted as correct. For the strict criterion scoring, only the exact same idea units were counted as correct, but different conjugations of the correct verbs and changes in the location of the idea units were also accepted. For the verbatim criterion scoring, the idea units were not used, and only the exact same sentences were counted as correct.

The interrater reliability for the independent raters was calculated for the lenient and strict criterion recall scores by using Pearson correlation. Results showed a strong significant positive correlation between the scores of the raters for the lenient criterion, *r*(86) = .99, *p* < .001, and for the strict criterion, *r*(86) = .99, *p* < .001. Minor differences in scoring were discussed among the raters and a compromise was reached for the final scoring. For the verbatim criterion, the scoring was first completed by the first rater and then checked by the second rater to ensure that only the same sentences were counted as correct.

### Analyses for the Recall and Recognition Tests

**Figure 2:**
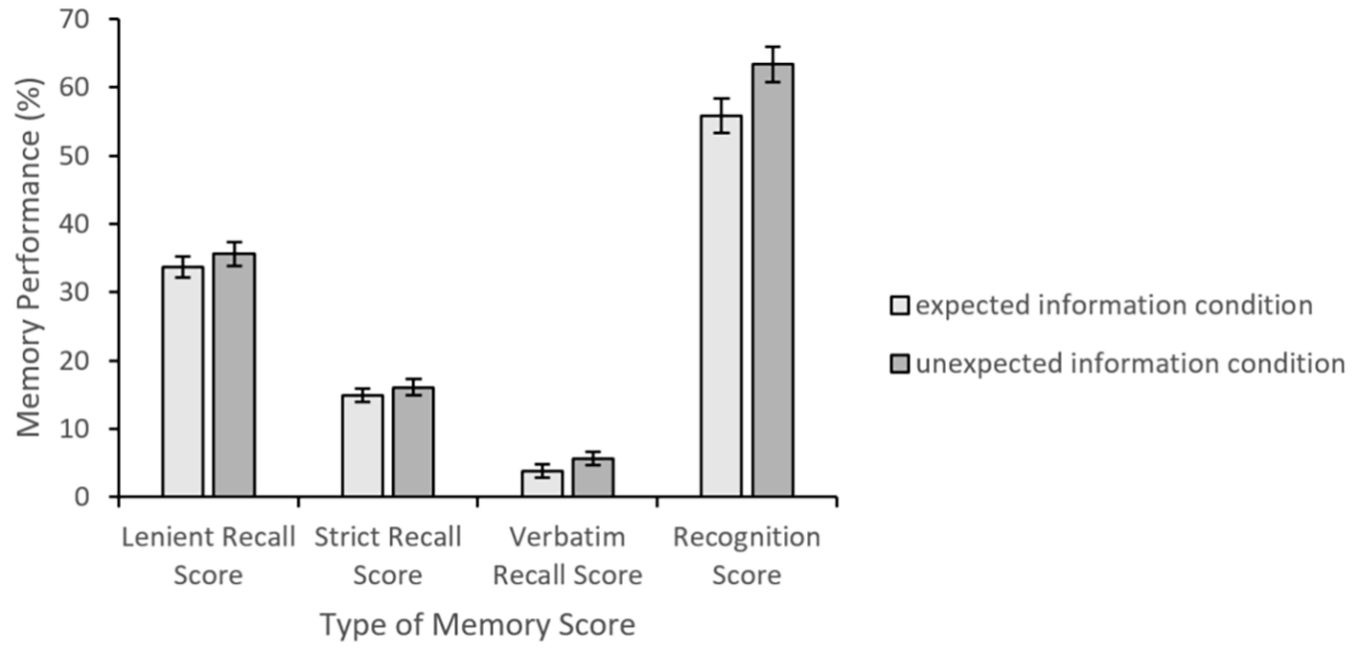
Mean Percent Recall and Recognition Scores *Note.* Error bars represent standard errors.

Percentages for the recall and the recognition test scores are presented in Figure 2.

Recall scores for the lenient criterion did not reveal a difference between the expected information (*M* = 33.71, *SE* = 1.52) and the unexpected information (*M* = 35.58, *SE* = 1.76) conditions, *t*(85) = 0.80, *p* = .43, *d* = 0.17. Similarly, there was no difference between the conditions for the strict scoring criterion, *M* = 14.91, *SE* = 0.99 for the expected information, and *M* = 16.06, *SE* = 1.21 for the unexpected information condition, *t*(85) = 0.73, *p* = .47, *d* = 0.16. The verbatim criterion for recall also revealed no difference between the conditions, *M* = 3.78, *SE* = 0.98 for the expected information and *M* = 5.68, *SE* = 1.03 for the unexpected information condition, *t*(85) = 1.34, *p* = .19, *d* = 0.29.

Recognition scores for the unexpected information condition (*M* = 63.35, *SE* = 2.65) were significantly higher than the expected information condition (*M* = 55.81, *SE* = 2.54), *t*(85) = 2.05, *p* = .04, *d* = 0.44.

In summary, the memory performance differences across the expected and unexpected information conditions were solely revealed in the recognition test.

### Analysis for the Recall and Recognition of the Misinformation

Pearson chi-square analysis was conducted to determine whether there were differences in the false recall and recognition of the misinformation. For the recall test, intrusions with the misinformation were minimal and similar in the two conditions: Three participants in the expected information condition (*n* = 43) and two participants in the unexpected information condition (*n* = 44) generated the misinformation in the recall test, χ^2^(1, *N* = 87) = 0.19, *p* = .66, *Cramer’s V* = 0.05.

For the recognition test, however, 24 participants in the expected information condition and 14 participants in the unexpected information condition falsely recognized the misinformation as accurate.^3^3There were two options that included the misinformation. Option A had an incorrect detail in the sentence along with the misinformation, whereas Option B was the correct sentence except for the misinformation. We report the percentage of participants who chose each of these options. Option D was the correct option. Expected information condition: Option A, 18.6%; Option B, 37.2%; Option C, 20.9%; Option D, 23.3%. Unexpected information condition: Option A, 11.4%; Option B, 20.5%; Option C, 13.6%; Option D, 54.5%. These frequencies were found to be distributed differently across the two conditions, χ^2^(1, *N* = 87) = 5.09, *p* = .03, *Cramer’s V* = 0.24. Thus, as hypothesized, participants in the unexpected information condition were less likely to recognize the misinformation as correct than the ones in the expected information condition.

### Exploratory Analyses for the Errors in the Recall Test

Further exploratory analyses were carried out to explore the type of errors in the recall test. Out of the 58 idea units in the passage, 10 critical idea units were selected that contained information about the characteristics (e.g., energetic, sporty), habits (e.g., going for a run every morning), actions (e.g., running after and catching the snatcher), and social life (e.g., having a crowded group of friends) of the main character. Depending on the condition, the idea units constituted either expected or unexpected information based on the age of the main character.

The number of omission, distortion, and commission errors were explored. An omission error refers to the absence of a critical idea unit. An omission error was recorded if the idea unit was absent in the scoring with the lenient criterion. A distortion error refers to a change in the content of the idea unit. A distortion error was recorded if the change in the idea unit was accepted as correct with the lenient criterion, but not with the strict criterion. Finally, a commission error refers to the addition of a new item to the passage. A commission error was recorded if an added item within the whole passage was not counted as correct even with the lenient scoring criterion. The commission errors were further divided and analyzed into three categories: 1) expected commission errors, 2) unexpected commission errors, and 3) unrelated commission errors. An expected commission error was recorded if the committed information was expected of the main character’s age based on stereotypes. An unexpected commission error was recorded if the committed information was unexpected of the main character’s age based on stereotypes. Unrelated commission errors were unrelated to the age of the main character.

Two independent raters scored errors. Pearson correlations showed strong correlations between the scores of the raters for the omission, *r*(85) = .96, *p* < .001, distortion, *r*(85) = .95, *p* < .001, and commission, *r*(85) = .91, *p* < .001 errors. Specifically, interrater reliability for the expected, *r*(85) = .89, *p* < .001, the unexpected, *r*(85) = .86, *p* < .001, and the unrelated, *r*(85) = .95, *p* < .001 commission errors was high. Minor differences in scoring were discussed among the raters and a compromise was reached for the final scoring.

Analysis for the omission errors did not reveal any difference between expected information (*M* = 5.00, *SE* = 0.25) and unexpected information (*M* = 4.77, *SE* = 0.28) conditions, *t*(85) = 0.60, *p* = .55, *d* = 0.13. There was also no difference across the conditions for the distortion errors. Specifically, *M* = 2.02, *SE* = 0.13 for the expected information, and *M* = 2.18, *SE* = 0.15 for the unexpected information condition, *t*(85) = 0.81, *p* = .42, *d* = 0.17.

**Figure 3:**
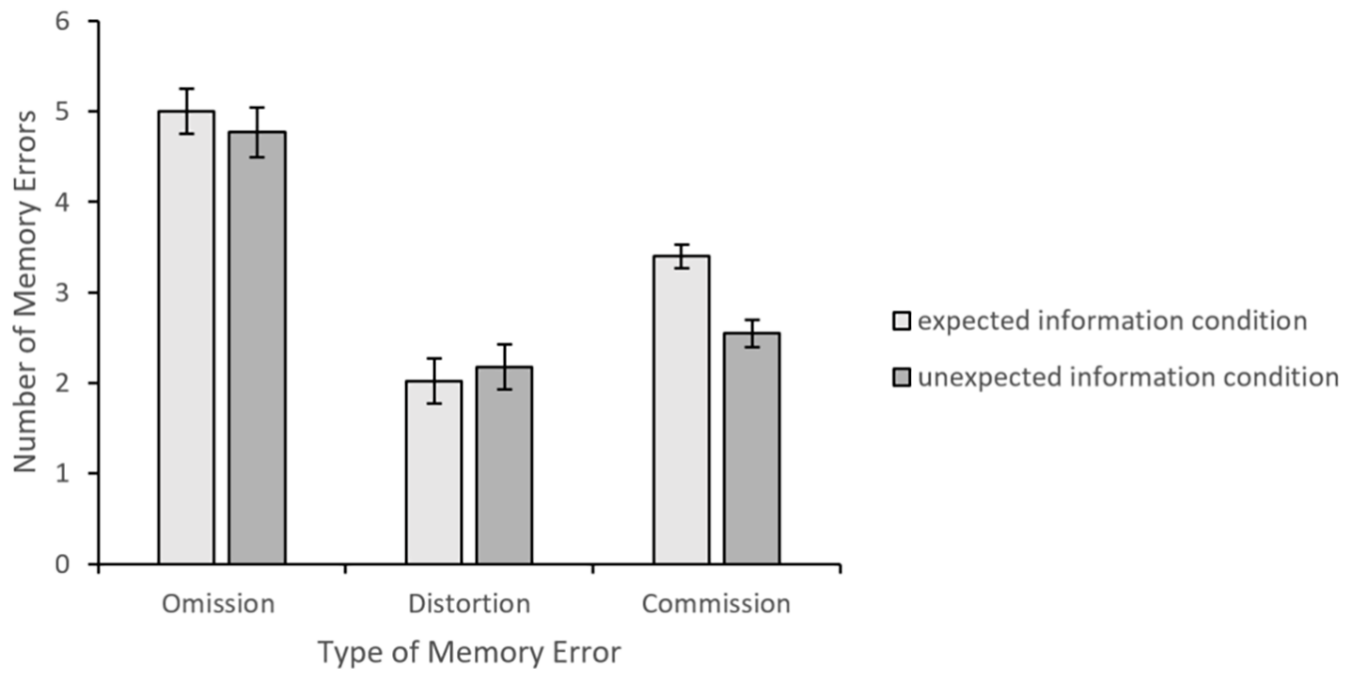
Mean Number of Memory Errors *Note.* Error bars represent standard errors.

There was a significant difference across the conditions for the overall commission errors. There were more commission errors in the expected information condition (*M* = 3.40, *SE* = 0.25) than in the unexpected information condition (*M* = 2.55, *SE* = 0.25), *t*(85) = 2.41, *p* = .02, *d* = 0.52. The results of the error analyses are presented in Figure 3.

**Figure 4:**
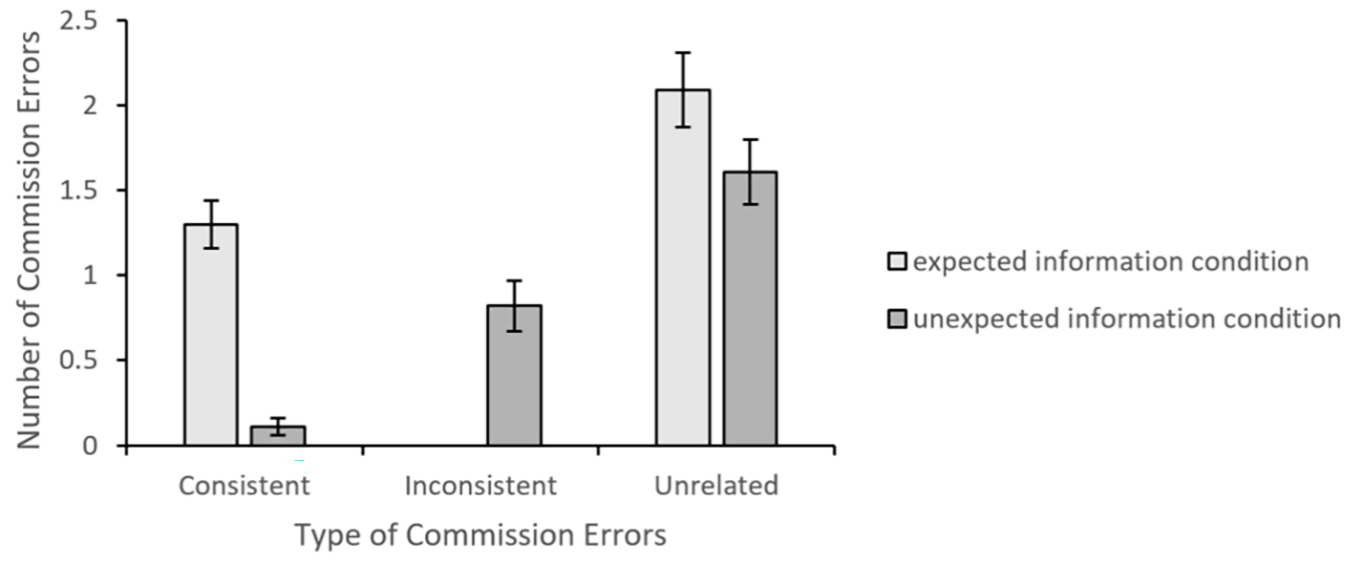
Mean Number of Commission Errors *Note*. Error bars represent standard errors.

A more detailed analysis of the commission errors showed that expected commission errors were more frequent in the expected information condition (*M* = 1.30, *SE* = 0.14) than in the unexpected information condition (*M* = 0.11, *SE* = 0.05) condition, *t*(85) = 7.93, *p* < .001, *d* = 1.70. On the other hand, unexpected commission errors were non-existent in the expected information condition and more frequent in the unexpected information condition *(M* = 0.82, *SE* = 0.15), *t*(85) = 5.52, *p* < .001, *d* = 0.17. The frequency of the unrelated commission errors did not differ across the expected information (*M* = 2.09, *SE* = 0.22) and unexpected information (*M* = 1.61, *SE* = 0.19) conditions, *t*(85) = 1.67, *p* = .10, *d* = 0.36. Thus, the commission errors were likely to be consistent with the information that was presented in the passage. The results of the analyses for the types of commission errors are presented in Figure 4.

## Discussion

In the present study, we aimed to investigate the effects of the expectedness of age-related information on memory performance, including false memories. We hypothesized better memory and less vulnerability to false memory when the information is stereotypically unexpected. The results partly confirmed our hypotheses by showing that the recognition of unexpected information was higher and that there was also lower false recognition of the misinformation when the information was unexpected based on age stereotypes. These results are consistent with the SP + T Model (Woll & Graesser, 1982), which predicts better memory for information that is unexpected, due to the more attentive processing it receives. Based on this view, we had predicted that prior knowledge can affect the extent of our attention to the information which, in turn, can affect memory accuracy and false memory formation. When there is expected information in a passage, it is likely that there will not be much processing for any of the information, possibly due to a sense of familiarity. On the other hand, if there is unexpected information in a passage, attention is likely to be necessary to process all information.

The recall of information and the false recall of the misinformation were not affected by whether the information was expected. The commission errors with stereotypically expected information in the recall test were higher when the information in the passage was stereotypically expected of the main character’s age. More specifically, as a novel finding of our research, commission errors were more likely to be expected commissions that are consistent with the age of the main character in the expected information condition and they were more likely to be unexpected commission errors in the unexpected information condition.

### Recall Tests Versus Recognition Tests

Our findings imply that the type of memory test (i.e., recall versus recognition) may be a critical factor related to how expected and unexpected information is processed.

Our recognition memory results are consistent with the notion that information that is expected is more likely to be retrieved as a gist (Araya et al., 2003; Brainerd & Reyna, 2010). Recognition relies on familiarity more than recall (Kintsch, 1970), and this may be a reason that the differences across conditions were only revealed in the recognition test. Given that only the minor details of the correct sentences were changed in the incorrect choices of the recognition test, a sense of familiarity was probably preserved, increasing memory errors. Considering that the misinformation in the study was not stereotypical, yet that it was more likely to be falsely recognized in the expected information condition, the misinformation was possibly represented as a gist in memory that is open to distortion in the context of stereotypically expected information.

A useful measure to support this explanation could have been collecting “remember”/“know” judgments. While a remember judgment indicates conscious recollection of information, a “know” judgment indicates just a sense of familiarity in the absence of conscious recollection (Tulving, 1985). Previous investigations showed more know judgments for schematic information than for non-schematic information (e.g., Neuschatz et al., 2002). If these measurements were collected, they could be useful in explaining the underlying process for the recognition results and in explaining the discrepant results for the recall test. Presumably, there would be more know judgments in the expected information condition than in the unexpected information condition. Know judgments would also be presumably higher for the recognition test than for the recall test (Wixted & Squire, 2010). This limitation of the present study can be overcome in future studies, by including these measurements.

The absence of differences across conditions in the recall test can also be explained by considering the nature of the two memory tests. Recall tests are usually more difficult than recognition tests. Recall tests involve two stages, but recognition tests involve only a single stage (Anderson & Bower, 1972; Kintsch, 1970). In recognition tests, the old information needs to be identified as being presented. On the other hand, in recall tests, the information needs to be retrieved first and then selected as the correct information among the retrieved items. Failure of either of these stages may result in reduced recall. In the present study, the difficulty of the recall test may have also limited the amount of information that is retrieved, making the scores in the two conditions more similar to each other. The performance of the participants on the recall test was 34% and 36% for the expected and unexpected information conditions respectively, even when the scoring criterion was lenient. In the recognition test, the retrieval performance was higher. Specifically, it was 63% in the unexpected information and 56% in the expected information conditions. Thus, both familiarity-based retrieval and retrieval difficulties may have contributed to the observation of the current findings.

Due to the null results that we obtained in the recall test, we further analyzed the types of errors that were made in the recall of the passages. The results showed that commission errors were higher in the expected information condition than in the unexpected information condition. Further analyses showed that commission errors were likely to be consistent with the information in the passage. Thus, stereotypically expected information may have been likely to receive attentive processing.

In general, our results indicate that an event description that involves stereotypical information may generally result in worse recognition and more false retrieval. Implications of these findings can be considered in eyewitness testimony research as well because they indicate that an eyewitness may be less accurate about the details of an event when the event details involve stereotypically expected information.

### Suggestions for Future Research

We also would like to consider some procedural details that can affect memory performance for stereotypically expected or unexpected information and make some suggestions for future research. Kleider and colleagues (2008) showed that information that is unexpected based on stereotypes was retrieved better in an immediate test, whereas expected information was retrieved better in the delayed test. Their immediate test was right after a 20-minute distractor task, while the delayed test was two days later. In our study, testing was done immediately after a 4-minute filler task, and our results were similar to Kleider et al. (2008) results for the immediate test. In future research, the time course of the retrieved information that is expected or unexpected based on age stereotypes can be investigated to gain insight into its persistence and recovery.

In the present study, we focused on age-related physical performance and social behavior stereotypes. Prior research shows that older adults internalize both physical and cognitive aging stereotypes. Future research can investigate the processing of age-related expected and unexpected information about cognitive performance by younger adults. Extension to aging stereotypes of cognition would provide information about the generalizability of the current findings.

There can also be some individual difference variables that can affect memory processes for stereotypically expected or unexpected information. One individual difference variable may be the prejudiced attitudes toward older adults. Swift et al. (2018) claimed that aging stereotypes can be both positive and negative. Negative stereotypes of aging are more dominant than positive ones (e.g., Kite et al., 2005; Kite & Johnson, 1988) and this escalates ageism (Greenberg et al., 2004). The two groups that were tested in the present study did not differ in their attitudes toward older individuals. Exploratory analyses also showed that the attitude measures did not correlate with memory measurements. Thus, individual differences in attitudes cannot be an explanation for the group differences that we found in recognition. Attitudes of our participants toward older adults were also quite positive. It should be noted though our attitude measures were taken explicitly. Nosek and colleagues (2007) collected online data about implicit stereotyping over six years. This large-scale data revealed a striking finding: The strongest implicit prejudice effect was observed for aging attitudes, where 80% of participants had difficulty in pairing *old people* with *good* items and *young people* with *bad* items. Moreover, these implicit aging attitudes were found to be cross-culturally consistent. Other studies also confirmed the automaticity of implicit negative age stereotypes (e.g., Krings, 2004). Our results also imply more automatic, i.e., less effortful and more error-prone processing of information that is expected based on age stereotypes. However, a systematic investigation of the relationship between implicit aging attitudes and memory measures can provide more direct evidence of the effects of individual differences in attitudes toward older adults.

Individuals may differ in the way that they handle stereotypical information as well. Need for cognition is defined as an individual’s tendency to engage in effortful cognitive activity and individuals who are high in need for cognition semantically process information in a deep and elaborative manner (Cacioppo et al., 1996). According to the fuzzy-trace theory, semantic processing leads to meaning-based gist traces, and the activation of semantic connections can lead to false memory (Brainerd & Reyna, 2002). Previous studies showed increased false memory among individuals who have high needs for cognition both in recognition (e.g., Graham, 2007) and in recall (e.g., Leding, 2011). Future research can investigate whether the findings can be generalized to both stereotypically expected and unexpected information.

Additionally, the need for cognitive closure as a motivational desire to resolve ambiguous, uncertain information that causes discomfort (Kruglanski & Webster, 1996) may also be an individual difference variable that might affect how an individual deals with unexpected information. The need for cognitive closure results in a search for stereotypically consistent information and the avoidance of inconsistent information in order to prevent any conflict with pre-existing schemas (e.g., Dijksterhuis et al., 1996). Further research can be carried out to determine the systematic effects of the need for cognitive closure in the retrieval and false retrieval of stereotypical information.

### Conclusion

The present study contributed to the literature on how aging stereotypes affect memory processes. The results show that stereotypically expected information is retrieved less accurately. Perpetuation of stereotypical ageist expectations hinders older adults’ performance and well-being (for a review see Chasteen & Cary, 2015). Therefore, the ultimate aim of research should be to reduce stereotypical processing. There are some promising studies pointing to factors that can decrease stereotyping such as motivation to generate accurate social impressions, taking the perspective of an out-group member, and being exposed to counter-stereotypical information (for a review, see Plous, 2003). It is important to understand the cognitive processes for the information that is expected based on stereotypes so that the findings in this line of research can eventually be useful in reducing prejudice and discrimination.

## Supplementary Materials

For this article, the following Supplementary Materials are available:

- Data. (Gülseren & İkier, 2025)
- Code. (Gülseren & İkier, 2025)
- Study materials. (Gülseren & İkier, 2025)

## Data Availability

For this article, data, codebook and materials are available at Gülseren and İkier (2025).
